# N6-methyladenosine RNA modification: an emerging molecule in type 2 diabetes metabolism

**DOI:** 10.3389/fendo.2023.1166756

**Published:** 2023-07-07

**Authors:** Haocheng Zhang, Yan Gu, Qiaojian Gang, Jing Huang, Qian Xiao, Xiaoqin Ha

**Affiliations:** ^1^ The Second School of Clinical Medicine, Lanzhou University, Lanzhou, Gansu, China; ^2^ Department of Clinical Laboratory, The 940th Hospital of Joint Logistics Support Force of Chinese People’s Liberation Army, Lanzhou, Gansu, China; ^3^ Key Laboratory of Stem Cells and Gene Drugs of Gansu Province, Lanzhou, Gansu, China; ^4^ College of Veterinary Medicine, Gansu Agricultural University, Lanzhou, Gansu, China; ^5^ School of Public Health, Gansu University of Traditional Chinese Medicine, Lanzhou, Gansu, China

**Keywords:** insulin resistance, metabolism, m6A modification, signaling pathway, type 2 diabetes

## Abstract

Type 2 diabetes (T2D) is a metabolic disease with an increasing rate of incidence worldwide. Despite the considerable progress in the prevention and intervention, T2D and its complications cannot be reversed easily after diagnosis, thereby necessitating an in-depth investigation of the pathophysiology. In recent years, the role of epigenetics has been increasingly demonstrated in the disease, of which N6-methyladenosine (m6A) is one of the most common post-transcriptional modifications. Interestingly, patients with T2D show a low m6A abundance. Thus, a comprehensive analysis and understanding of this phenomenon would improve our understanding of the pathophysiology, as well as the search for new biomarkers and therapeutic approaches for T2D. In this review, we systematically introduced the metabolic roles of m6A modification in organs, the metabolic signaling pathways involved, and the effects of clinical drugs on T2D.

## 1 Introduction

Type 2 diabetes (T2D) is a chronic metabolic condition characterized by high blood glucose levels, insulin resistance (IR), and insulin secretion deficiency. Over the past two decades, the number of people living with diabetes has more than tripled, from 151 million in 2000 to 537 million in 2021 (1). Thus, diabetes is becoming one of the fastest-growing metabolic disorders of the 21^st^ century. T2D is the most common type worldwide, constituting about 90% of all diabetes cases (2). In addition to the rising number of people suffering from the disease, there has been a dramatic increase in the costs to healthcare systems and individual financial burdens due to T2D (3). Nonetheless, since the detection of the sequence of the human genome for the first time in 2001 (4), we gained new insights into the development of diseases with the development of omics. Based on these technologies, the pathogenic mechanisms underlying the rising prevalence of T2D can be explored further for refined phenotyping, prevention and treatment.

Epigenetics has been defined as reversible and heritable changes in gene functions without altering the DNA/RNA sequence that comprises DNA/RNA methylation, histone modifications, and noncoding RNA-mediated processes (5). Among various post-transcriptional RNA modifications, N^6^-methyladenosine (m6A) is the most abundant modification on RNA molecules and the most prevalent methylated nucleoside presented in eukaryotes (6). The m6A modification plays a biological role regulated by “writers” methylases, “erasers” demethylases, and “readers” TYH domain families (7). Several studies have shown that m6A can bind to mRNA and affect the expression of target genes, thus regulating various physiological molecular mechanisms, such as cell cycle, energy metabolism, and inflammation (8). Importantly, m6A modification can respond to changes in homeostasis of the internal environment, which might regulate the sustained long-term expression of T2D-related pathogenic genes induced by prior hyperglycemia exposure (9). This mechanism could explain the early pathogenesis of T2D. In the present review, we describe the perspectives and the role of m6A modification in T2D and discuss the novel insight into the pathogenesis of T2D that could be translated into novel biomarkers and therapeutic modalities.

## 2 The m6A modification

Post-transcriptional RNAs can be modified by more than 170 diverse modifications analogous to various biomacromolecules (10). Several mRNA modifications, including m6A, 5-methylcytidine (m5C), and N^1^-methyladenosine (m1A), have been reported based on a transcriptome-wide mapping approach (11). Among this, m6A is presently the most common modification in mammalian mRNA and long non-coding RNA, which account for more than 50% of eukaryotic methylation modification, and approximately 0.1%-0.4% of all adenosines have m6A modification (12). The m6A modification has the highest distribution at the 3’ end of the transcripts, near the end of coding regions and at the last exon of the non-coding regions. Specifically, its main manifestations are G-(m6A)-C (70%) and A-(m6A)-C (30%) (13), whose distribution on RNA is 37%: 28%: 20%: 12%: 3% for coding sequence: stop codon: 3’-untranslated region: transcription stop site: 5’-untranslated region (14). The m6A modification plays a vital role in modulating RNA processing, degradation, and stability, catalyzed by a multi-component enzyme complex (also called “writers”), reversed by demethylases in the nucleus (termed as “erasers”), and regulated by YTH domain family (also known as “readers”) (7). Accumulating evidence has shown that the regulation of target genes by m6A modification and its effect in T2D depends on two factors: I. The abnormal level of m6A modification in T2D mainly depends on the expression and activity of “writers” and “erasers.” II. Targets are critical genes affecting glucose and lipid metabolism, insulin secretion, and IR.

To date, most of the studies have focused on “writers.” The most decisive part of m6A modification “writers” is a methyltransferase complex, composed of METTL3, METTL14, WTAP, and VIRMA (KIAA1429). METTL3 and METTL14 form a heterodimer, which is the main methylase catalyzing the transfer of S-adenosyl methionine (SAM) to bind to specific RNA sites, a well-conserved motif DRACH (D = G/A/U, R = G/A, H = A/U/C) (15). WTAP is a critical regulatory subunit of the complex, recruiting METTL3/14 heterodimer into nuclear speckles and promoting its RNA binding ability. VIRMA is the complex’s main scaffold that promotes RNA anchoring and is involved in mRNA polyadenylation (16). In addition, other “writers” consist of RBM15/15B, ZC3H13, ZCCHC4, METTL16, METTL4, and METTL5. RBM15/15B and ZC3H13 interact with WTAP, promoting the complex catalytic function (17). ZCCHC4 is mainly associated with the methylation of human 28S rRNA (18). METTL16 differs from METTL3/14 due to its interaction with eIF3 and rRNAs to facilitate methylation (19). METTL4 is mainly related to m6A modification in internal U2 snRNA (20), and METTL5 is associated with m6A at adenosine 1832 of 18S rRNA (21).

Compared to “writers,” the components of demethylase “erasers,” FTO and ALKBH5, are much simpler. Both belong to the ALKB family of non-heme Fe(II)dioxygenases, and the former is not only involved in removing m6A modification but also contributes to human obesity (22), whereas the latter is a crucial demethylase located in nuclear-nascent RNAs, with maximal expression in the testis, heart, and kidney (23).

**Figure 1:**
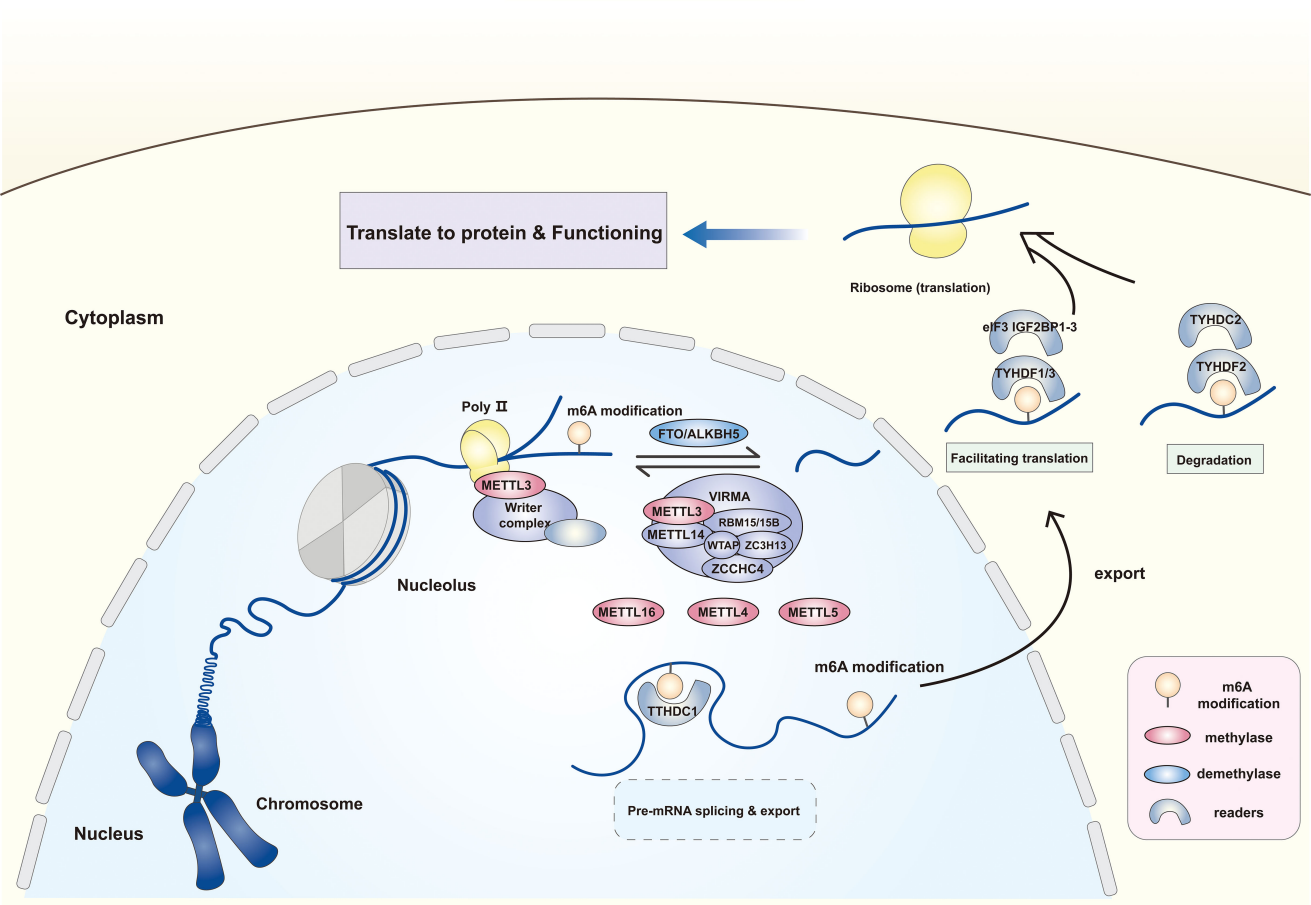
The expression location and form of function of m6A methylases, demethylases and readers. The m6A modification of RNA is dependent on the catalytic effect of methylases and demethylases. The former mainly includes methyltransferase complex with METTL3 as the core, METTL16, METTL4 and METTL5. The latter comprises the FTO and AKLKBH5. The m6A site on RNA can be recognized and combined by different “readers” to influence the RNA following effects by facilitating translation, splicing and affecting stability. METTL3/14/16/5/4, Methyltransferase-like 3/14/16/5/4; WTAP, Wilms tumor 1- associated protein; VIRMA, Vir-like m6A methyltransferase associated; RBM15/15B, RNA binding motif protein 15/15B; ZC3H13, ZCCHC4, zinc finger CCHC domain-containing protein 4; FTO, Fat mass and obesity-associated protein; ALKBH5, AlkB homologue 5; eIF3, Eukaryotic translation initiation factor 3; YTHDC1/2, YTH domain containing 1/2; YTHDF1-3, YTH N6-methyladenosine RNA binding protein 1-3; IGF2BP1-3, Insulin-like growth factor 2 mRNA binding protein 1-3.

In addition to “writers” and “erasers” that can directly methylate/demethylate RNAs, “readers” recognize and combine the m6A modification, which includes the YTHDF family, TYHDC family, IGF2BP family, hnRNPs, FMRP, eIF3, and PRRC2A. Among these, the star molecules, TYHDF and THYDC families are widely studied. The TYHDF family entails TYHDF1–3, of which TYHDF2 effectuates target transcript degradation through the deadenylation pathway or the endoriboncleolytic pathway, while TYHDF1/3 has opposite functions, binding to eIF3 to facilitate the translation of target transcripts (24). TYHDC1, a TYHDC family protein, could anchor to the m6A modification of mRNA and mediates mRNA splicing, but this effect is currently found only in Drosophila (25). In addition, it accelerates nuclear mRNA export by binding to the SR protein family (26). While YTHDC2 is involved in mRNA degradation and translation initiation (27). The ribosomal proteins, include the IGF2BP family and FMRP, also function in m6A modification, strengthening mRNA stability (28) (**Figure 1**).

To date, the detection methods for RNA modification are mainly based on mass spectrometry (MS) and high-throughput sequencing techniques. The former tends to be detected using liquid chromatography-MS (LC-MS) or tandem mass spectrometry (LC-MS/MS), which can detect the overall m6A abundance of mRNA and has high sensitivity and specificity as its detection is based on the physicochemical properties of m6A modification such as molecular mass and chromatographic retention time. However, it has higher requirements for sample handling, and if the sample has components of other RNAs, the source of m6A modification cannot be determined (29). In addition, LC-MS or LC-MS/MS does not provide sequence and localization information (28). High-throughput sequencing technology is another common method for m6A modification detection, which can determine the presence of mRNA m6A modification and its specific location in the transcriptome. But this approach relies on anti-m6A antibodies, which makes it impossible to obtain high-resolution information about the loci (30).

## 3 The m6A modification and organ metabolism in T2D

**Figure 2:**
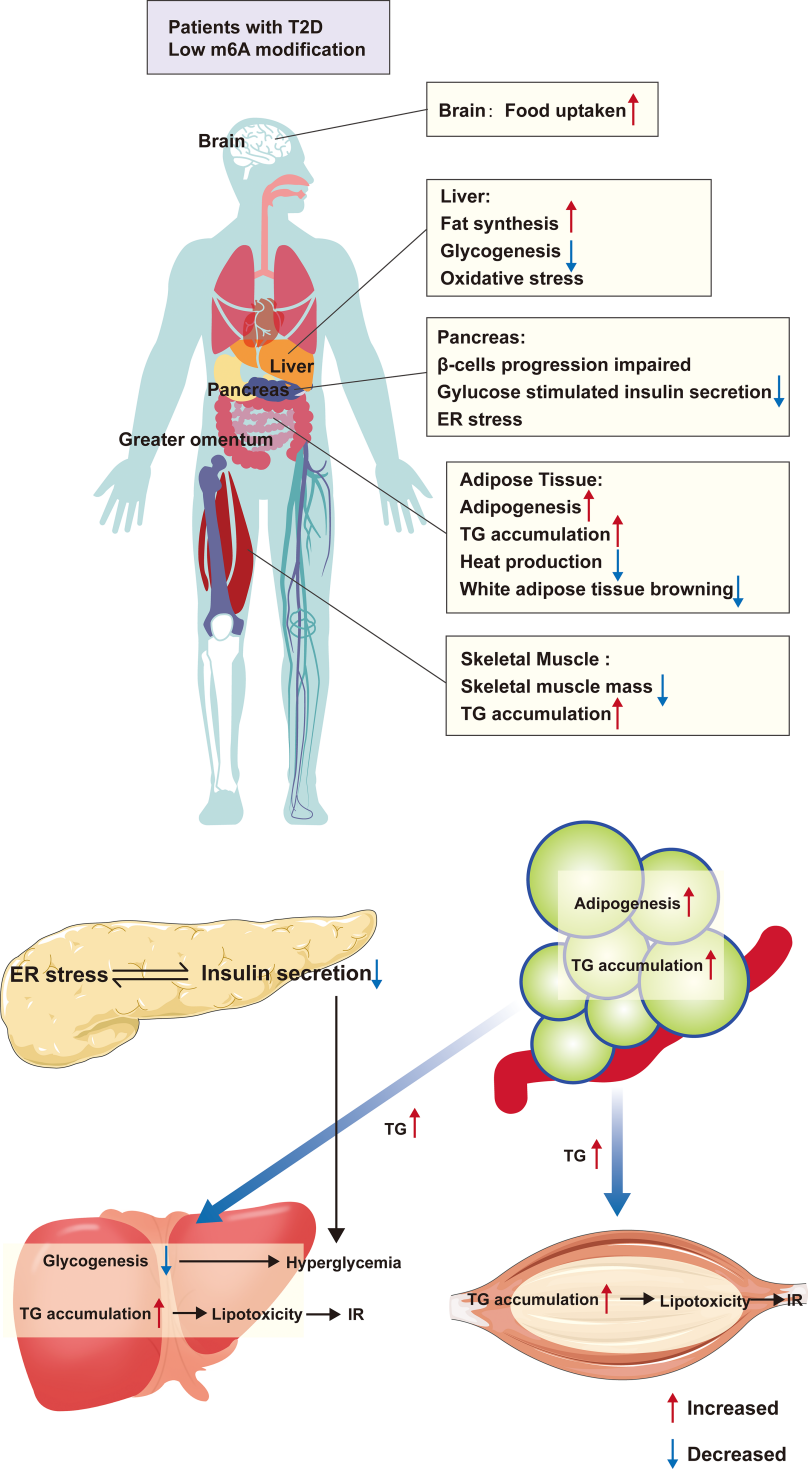
The m6A modification and organ metabolism in T2D. Patients with T2D are in a condition of low m6A modification, which causes functional abnormalities in multiple organs throughout the body. Firstly, in adipose tissue, there is excessive lipid droplet accumulation, increased white adipose tissue and decreased browning, which causes the release of large amounts of TG from adipose tissue into the circulation, and these TG absorbed by the liver and skeletal muscle, resulting in inflammation and IR due to lipotoxicity. Furthermore, the condition also directly affects the function of islet β-cells, leading to abnormal differentiation and ER stress, all of which cause a decrease in insulin secretion In addition, low m6A modification alters the expression of hepatic glycolipid genes, leading to hepatic steatosis as well as reduced glycogen synthesis. All of these further aggravates the hyperglycemic symptoms in T2D patients. ER, endoplasmic reticulum; TG, triglycerides; IR, insulin resistance.

Typically, the development of T2D is the combination of impaired pancreas islet cells and metabolic disorders. Various causes of islet cell damage decrease the β-cell mass, leading to insulin deficiency. On the other hand, a massive accumulation of energy into adipose tissue, especially white adipose tissue, upregulates the adipose inflammatory factors, following which fat metabolites are transported into the liver and skeletal muscle leading to IR, further aggravating the metabolic disorder. These inflammatory factors also lead to islet cell stress, further damaging the islet cell biology (31). Therefore, we first described the role of m6A modification in the four major target organs of T2D (**Figure 2**).

### 3.1 Pancreas

As a response to endogenous or exogenous stimuli, such as glucose, lactose, and glucagon, the pancreatic β-cells release insulin, the only hypoglycemic hormone in the body. The loss of islet β-cell function is the decisive pathological mechanism leading to T2D (32). Accumulating evidence revealed that m6A modification is involved in β-cell dysfunction.

An *in vivo* study on the function of METTL14 in islet β-cells demonstrated that β-cell specific-knockout of *Mettl14* mice leads to β-cells death and abnormal β-cell differentiation, contributing to low β-cell mass and insulin secretion (33). Another study (34) showed that EndoC-βH1 cell with *Mettl3/14* knockdown induced G0/G1 cell cycle arrest and decreased insulin secretion. They also analyzed m6A hypomethylation-mediated transcription *in vivo* and *in vitro*, which mainly involved the cell cycle regulatory genes and insulin/insulin-like growth factor 1-serine/threonine kinase-pancreatic and duodenal homeobox 1 (insulin/IGF1-AKT-PDX1) pathway. Deleting *Mettl3/14* decreased the insulin-induced AKT phosphorylation, resulting in low PDX1 expression, a vital transcription factor to maintain β-cells biology (35). Men et al. (36) further supplemented the role of METTL14 in β-cell biology. The results indicated that genes responsible for endoplasmic reticulum (ER) stress were upregulated in *Mettl14* knockout islet β-cells, especially *ER to nucleus signaling 1* (*Ern1/Ire1α*) and *X-box protein binding 1* (*Xbp-1*). The former could respond to ER stress and unconventionally activate the latter mRNA, resulting in the overexpression of degraded proteins in the ER and slicing insulin mRNA (37). Wang et al. (38) reported that if endocrine progenitors lost Mettl3/14, they mature toward β-cells defectively, leading to early hyperglycemia. Moreover, the mechanism showed that loss of *Mettl3/14* downregulates *MAF bZIP transcription factor A* (*Mafa*) mRNA stability. Interestingly, the abundance of MAFA protein decreased, and glucose-stimulated insulin secretion (GSIS) impaired the preceded loss of β-cell mass. Both studies observed m6A modification does not cause IR and rarely alters insulin sensitivity, indicating that dynamic m6A modification following internal environmental changes is involved in the early unknown pathophysiological development of T2D in the pancreas. This hypothesis could also be confirmed by the findings of Li et al. (39). Under inflammation and oxidative stress conditions, METTL3 level decreased in islet cells, and islet β-cell deficiency of *Mettl3* further induces hyperglycemia. On the other hand, methylglyoxal (a precursor of the advanced glycation end product) treatment of β-cells downregulated the METTL3 level, affecting MAFA mRNA and protein levels and decreasing GSIS (40).In addition, a study illustrating the lack of WTAP in the T2D pancreas deserves our attention (41). Deficiency of *Wtap* in pancreas decreases the functional stability of MEETL3 protein, leading to pancreas hypomethylation and dysfunction. This implies that exploring other non-catalytic enzymes in the methylesterase complex to influence m6A levels might be another effective therapeutic option.

“Erasers” are also involved in β-cells. Especially, FTO is an obesity gene but also partakes in the mRNA m6A modification of other core proteins. Several cohort studies (42–45) demonstrated that many variants of *Fto* and *Igf2bp2* are related to an upregulated risk of T2D occurrence, and both are involved in islet β-cell biology. However, the studies on the role of FTO are controversial and have completely opposite results. Interestingly, the *Fto* knockdown *in vitro* inhibited insulin secretion, which was interpreted as FTO-enhancing β-cell exocytosis to increase the first phase insulin secretion rather than only enhancing insulin biosynthesis (46). Another recent study (47) indicated that silenced *Fto* stimulates glucose-induced insulin secretion by upregulating the gene expression maintaining the identity of β-cells, such as *paired box 4* (*Pax4*), *glucokinase* (*Gck*) and *solute carrier family 2 member 2* (*Slc2a2*/*Glut2*). In addition, the study reported that m6A modification is reduced in human T2D islets through the upregulated expression of *Fto* and *Albhk5* mRNA and revised subcellular localization into the major m6A modification sites. In comparison with the essential functions of “writers” and “readers” in pancreatic growth/maturation/function, “erasers” in the pancreas seems to be more like an auto-regulator, which only alters the m6A modification of insulin secretion-related genes.

Similarly, “readers,” the major component of m6A RNA modification, also play a critical role in β-cell biology. Regue et al. (48) reported that mice with *Igf2bp2* deletion showed decreased leanness, increased energy consumption, and reduced insulin secretion. Mechanically, the IGF2BP2 binding site is the *Pdx1* mRNA m6A modification site, which improves the stability of *Pdx1* mRNA, facilitating its translation. A cross-sectional study from China also verified that IGF2BP2 and IGF2BP3 were elevated in pancreatic samples from T2D patients (49). Additionally, TYHDC1 is also critical for pancreas function. TYHDC1 is significantly downregulated in T2D individuals, and lack of *Tyhdc1* in β-cells leads to chronic inflammation, insulin secretion damaged and hyperglycemia (50). In a previous review (51), Dai concluded that the association between IGF2BP2 single nucleotide polymorphisms (SNPs) and T2D primarily concentrated on β-cell function deletion rather than reduced insulin sensitivity.

Accumulating evidence showed that the m6A modification components in β-cells might contribute to the pathophysiology of T2D before its onset, while the mRNA hypomethylation characteristics of genes in the insulin/IGF1-PDX1 pathway in T2D islets might be novel targets for the early diagnosis of T2D.

### 3.2 Adipose tissue

In addition to β-cell biology dysfunction and progressive insulin secretion deficiency, IR is a critical pathophysiological mechanism in T2D. Some studies suggested that the development of IR is closely associated with adipose tissue. Hyperglycemia contributes to the bioconversion of glucose to triglycerides (TGs) that accumulate in the white adipose tissue. Excessive energy accumulation in adipose tissue triggers the release of free fatty acids (FFAs) transported to the liver and skeletal muscle, leading to IR (52). On the other hand, inflammatory factors of adipose tissue reduce insulin sensitivity in the liver and skeletal muscle and aggravate IR (53). Accumulating evidence demonstrated that an essential role of m6A modification in stem cell transformation into preadipocytes and preadipocytes differentiation into mature adipocytes (54).

Yao et al. (55) demonstrated that METTL3 and TYHDF2 are essential for bone marrow stem cell adipose differentiation. Furthermore, *Mettl3* silencing enhances the expression of janus kinase 1/signal transducer and activator of transcription 5/CCAAT enhancer binding protein beta (JAK1/STAT5/C/EBP β) pathway and mRNA levels of *peroxisome proliferator-activated receptor gamma* (*Pparγ*), *CCAAT enhancer binding protein alpha* (*C/ebpα*), and *fatty acid binding protein 4* (*Fabp4*) in adipogenesis. Mechanistically, si*Mettle3* decreases the methylation abundance on *Jak1* mRNA, reducing the degradation of TYHDF2 due to m6A sites reduced in *Jak1* mRNA and upregulating JAK1 protein expression and STAT5 phosphorylation, which then bind to C/EBPβ promoter to promote adipogenesis. Moreover, an enrichment analysis showed that developmental genes were enriched in Mettl3 and Mettl14 targets (56). Mitotic clonal expansion (MCE) is a mandatory process in adipogenesis; a previous study reported that WTAP recruits METTL3/14 heterodimer to regulate adipocyte differentiation (57). In another study (58), 3T3-L1 cells with si*Mettl3* showed that *cyclin D1* (*Ccnd1*), a conserved cell cycle gene, reduced the m6A modification level to avoid TYHDF2 degradation. Wang et al. (59) reported that knockout of *Mettl3* aggravated high-fat diet (HFD)-induced IR and obesity. An *in vitro* experiment showed that large lipid droplets accumulate in *Mettl3*-knockout cells via suppression of the mRNA and protein expression of uncoupling protein 1 (UCP1), PPARG coactivator 1 alpha (PCG-1α/PPARGC1A), PPARγ, and PR/SET domain 16 (PRDM16). A recent study on IR in obstructive sleep apnea syndrome showed that downregulation of *Mettl3* increased the TG levels, which released FFAs, upregulated glycerol, inhibited glucose utilization in the other organs, and induced IR (60). Since the IR induced by obstructive sleep apnea syndrome is similar to the IR pathogenesis in T2D, this study was included in our review.

Demethylase, especially FTO, plays a crucial role in adipogenesis and adipose metabolism. In a previous study, Merkestein et al. (61) reported that *Fto* deficiency on adipogenesis obstructed the MCE process by attenuating the short isoform of RUNX1 partner transcriptional co-repressor 1 (RUNX1T1), suppressing the levels of cell proliferation and cell cycle genes, such as *Ccnd1*, *Ccnd3*, *Fabp4*, and *C/edpα* during MCE process. RUNX1T1 has two functionally distinct spliceosomes comprising short and long isoforms; expression of the former enhances, whereas expression of the latter impairs adipogenesis (62). In addition, in obese individuals, TG flow causes macrophage to aggregate in adipose tissue, leading to chronic inflammation and facilitating IR. And FTO upregulates oxidized low density lipoprotein-induced AMP-activated protein kinase (AMPK) activation in macrophages to enhance TG flowing (63). Adipose tissue inflammation is an essential factor contributing to IR, in which macrophages play an important role. It will be interesting to explore how m6A modification involves in the biology of macrophages in lipid metabolism. A previous study on mice with adipose-specific knockout of *autophagy-related 5* (*Atg5*) and *Atg7* showed that the effect of anti-IR and anti-obesity could effectively regulate fat mass via autophagy (64). Furthermore, the study found that FTO overexpression significantly enhanced the levels of *Atg5/7* mRNA and promoted adipogenesis and TG accumulation by modulating the ATG5/7-C/EBPβ pathway. In another study (65), *Fto* deletion elevated the browning of white adipose tissue, promoting the conversion of TG accumulation in white adipose tissue into energy consumption in brown adipose tissue. Subsequently, the results suggested that FTO increases the m6A modification sites of hypoxia-*inducible factor 1 subunit alpha* (*Hif1-α*) mRNA, which is later discerned by YTHDC2 that promotes protein translation. In a recent review by Azzam et al. (66), the overexpression of FTO was shown to upregulate CCND1, CCND3, PPARγ, and C/EBPα in adipocytes to enhance adipogenesis, as well as regulate the skeletal muscle lipid droplet accumulation by attenuating the AMPK pathway. *Fto* is also identified as the strongest factor associated with obesity by the first genome-wide association studies (GWAS); it plays a remarkable role in adipogenesis. The SNPs in the first intron of the gene were significantly related to the risk of obesity and T2D (67–69). There are two debates about how *Fto* SNPs increase the risk of these diseases: I. *Fto* SNPs increase the expression of FTO, and it acts as an auto-regulator function to promote lipid metabolism genes in an m6A dependent manner (as we mentioned above), thus causing body adipogenesis (66, 70). II. *Fto* SNPs regulate promoter of other genes to function, such as *iroquois homeobox 3 and iroquois homeobox 5 (* 71). Ghrelin is an appetite stimulating hormone and its acylation is essential for appetite stimulation. *Fto* rs9939609 increases the expression of FTO, and increased the mRNA of ghrelin in an m6A-dependent manner, and increased the expression of acyl-ghrelin (72).

The studies on “readers” are widely scattered. YTHDF1 overexpression is related to enhanced adipogenesis (73), while YTHDF2 degrades the corresponding genes’ mRNA that recognize the m6A modification sites affected by methylases and demethylases (55, 58); only YTHDF3 has been reported to be associated with obesity (74). TYHDC2 increases the stability of *Hif1-α* mRNA to promote its expression (65). GWAS found that IGF2BP2 is functionally enriched in energy expenditure and FFA oxidation (75) and is associated with obesity-susceptible T2D (76).

Although studies on the role of m6A modification in T2D adipose tissue have been rarely reported, we speculated its role based on the low m6A modification level in T2D patients (77). T2D is closely associated with white adipose tissue, and adipose tissue hypomethylation level further leads to adipocyte differentiation and proliferation, which promotes FFA access to the liver and skeletal muscle, leading to IR and pre-T2D. Thus, whether hyperglycemia in the body leads to hypomethylation and obesity or whether obesity alters the methylation degree needs further investigation, which would improve our understanding of the pathogenesis of T2D.

### 3.3 Liver

The liver is one of the main metabolic organs that controls the metabolic homeostasis of the human body. Therefore, the metabolic disorders in the liver, especially the glucose and lipid metabolism disorders, are related to the pathological mechanism of T2D (78). Current studies have demonstrated that hepatic glucose and lipid metabolism disorders involved m6A modification.

In a previous study, Xie et al. (79) reported that m6A modification and METTL3 were upregulated in the hepatic tissues of T2D patients and positively related to homeostatic model assessment (HOMA)-IR. In addition, hepatocyte-specific knockout of *Mettle3* in high-fat diet (HFD)-fed mice improved insulin sensitivity and decreased fatty acid synthesis, which was mechanistically related to METTL3 targeting *fatty acid synthase* (*Fasn*) mRNA and increasing its stability. The role of METTL3 was supplemented in the study by Li et al. (80). They found that adeno-associated virus (AAV)-mediated hepatocyte-specific overexpression of *Mettle3*, which upregulated the HFD-induced liver metabolic disorders and IR. Furthermore, the mechanism showed that *Mettl3* deletion advanced the mRNA half-life of the primary regulators of liver metabolism, such as *Lpin1* and *Lpin2*. The inhibition of m6A modification via *Mettl3* knockdown decreases *Pparα* mRNA m6A abundance and expression of *sterol regulatory element-binding proteins-1c* (*Srebp-1c*), *Fasn*, and *acetyl-CoA carboxylase* (*Acc*) mRNA and increases *Pparα* mRNA half-life and expression, thereby reducing lipid accumulation and alleviating IR in HepG2 cells *in vitro (* 81). Yang et al. (82) innovated a new T2D mice model by specifically knocking out Tmeme30a in pancreatic islet β-cells and found that METTL3 and METTL14 were upregulated *in vivo*. Furthermore, the overexpression of METTL14 or METTL3 led to increased protein and mRNA levels of ATP citrate lyase (ACLY) and stearoyl-CoA desaturase 1 (SCD1) *in vitro (* 83). In the inorganic arsenic (iAs)-induced T2DM, iAs treatment promoted translocation of METL14 and IGF2BP2 to the nucleus and activated m6A-mediated (METTL14-mediated) NOD-like receptor protein 3 (NLRP3) inflammasome. Moreover, METTL14 and IGF2BP2 enhanced the stability of *Nlrp3* mRNA. Ultimately, iAs treatment induces early hepatic IR via m6A modification (84). Qin et al. (85) reported that METTL3 expression is elevated in obese mice hepatic macrophages, which would reduce *DNA damage inducible transcript 4* (*Ddit4*) mRNA level in an m6A-dependent manner. Downregulated *Ddit4* enhances the activation of mTORC1 and NF-κB signaling pathways, affecting hepatic oxidative stress, adipogenesis and glucose metabolism. Additionally, it has been shown that the high-glucose environment in the uterus of gestational diabetes changes the fetal epigenetic modification, making the generations more susceptible to T2D. Mechanistically, upgraded RBM15 inhibits the activation of insulin signaling pathway by regulating *Claudin 4* in an m6A manner (86). Although all of the above studies showed elevated levels of hepatic methylase, the current view is generally that this is attributed to the possibility that some unclear feedback mechanism in the liver regulates the levels of methylase and demethylase. When high blood glucose-stimulated increases in *Fto* leads to low m6A modification in the liver, and as a feedback, methylase levels rise to maintain normal m6A abundance (77). However, an alternative explanation suggested that a high level of oxidative stress leads to increased m6A modification (81).

Yang et al. (77) demonstrated that the level of *Fto* mRNA in T2D patients was higher than that in healthy individuals and was positively correlated with fasting blood glucose. Furthermore, *Fto* knockout in high glucose-treated HepG2 cells showed some mRNA of glycolipid metabolism genes downregulated, such as *forkhead box O1* (*Foxo1*), *glucose-6-phosphatase catalytic subunit* (*G6pc*), *diacylglycerol O-acyltransferase 2* (*Dgat2*), and *Fasn*. The overexpression of *Fto in vitro* also upregulated the mRNA levels of these genes. As an inhibitor of FTO, Entacapone reduces fasting blood glucose and affects gluconeogenesis and fat thermogenesis in diet-induced obese mice by acting on the FTO-FOXO1 regulatory axis (87). In this study, the treatment of obese mice with FTO inhibition improved hepatic glucose tolerance and reduced white adipose tissue production. Considering that m6A methylase is essential for physiological function of the pancreas and that drug agonists are also more difficult to find than antagonists, therefore, FTO may be a better drug target for T2D. A recent study (88) showed that FTO affects *sterol regulatory element binding transcription factor 1* (encode SREBP-1C) and *carbohydrate responsive element binding protein*, two adipogenesis transcription factors, enhancing lipid accumulation in a m6A-dependent manner. Since the role of FTO in the liver is not limited to glucose and lipid metabolism, Lim et al. (89) focused on the correlation between FTO and inflammation. The results demonstrated that *Fto* knockout relieved palmitic acid (PA)-induced oxidative stress, mitochondrial dysfunction, ER stress, and apoptosis *in vitro*. The high expression of FTO in T2D liver might be due to the high glucose environment stimulating insulin over-secretion. The binding of insulin to the insulin receptor in the liver causes insulin receptor (INSR) β to translocate to the nucleus and bind to the promoters of target genes, including *Fto (* 90). This study suggests that hyperglycemia is a direct factor of hypomethylation in T2D patients, but the biology of this effect in IR still needs further investigation. Interestingly, in the liver both methylesterase and demethylases are most important in regulating lipid metabolism rather than carbohydrate metabolism. Nonetheless, the remaining demethylases, such as the ALKBH family, are poorly reported with T2D.

Zhou et al. (91) reported that TYHDC2 was significantly downregulated in the liver of obese mice, which resulted in the accumulation of hepatic TGs. Subsequently, *Tyhdc2* overexpression *in vivo* ameliorated hepatic steatosis and IR. For further verification, ob/ob mice were injected with YTHDC2, which significantly lowered the blood glucose in fed/fasted states and improved the performance in the insulin tolerance test. The underlying mechanism is that YTHDC2 binds to the mRNA of lipogenic genes, including *Srebp-1c*, *Fasn*, *Scd1*, and *Acc1* to decrease their mRNA stability and inhibit gene expression. Some studies have reported that YTHDF2 and eIF3G are upregulated in T2D model mice (83), high-glucose-induced HepG2 cells had upregulated *Ythdf1* mRNA (92), and high level of ROS *in vivo* can increase the level of *Ythdf2* mRNA (81), but there is no explanation for these results.

### 3.4 Skeletal muscle

Skeletal muscle is a critical organ for maintaining the homeostasis of glucose metabolism throughout the whole body, and 80% of postprandial blood glucose regulation depends on skeletal muscle. Therefore, IR in skeletal muscle is a significant disease mechanism in T2D (93).

A past review (94) demonstrated the presence of accelerated loss of skeletal muscle in patients with T2D and a significant difference in total skeletal muscle mass between patients with and without T2D. A study of m6A modification and skeletal muscle growth *in vivo (* 95) reported that METTL3 upregulation and increased m6A abundance implied physiological hypertrophy of skeletal muscle; the molecule is also required to maintain normal skeletal muscle function and muscle mass. Mechanistically, METTL3 regulates the abundance of methylation on mRNA of an activin receptor, *activin type 2A receptor* (*Acvr2a*), which is then recognized by YTHDF2 and degraded. As one of the negative regulators of skeletal muscle (96), ACVR2A phosphorylates SMAD3 protein, exerting anti-skeletal muscle hypertrophy effects. Similarly, another review concluded that METTL3/14 positively regulates skeletal muscle proliferation and differentiation (97). In the case of T2D, the mass of skeletal muscle and the IR of skeletal muscle are the major defects. Huang et al. (98) reported that PA-induced C2C12 myotubes resulted in IR-mediated reduction in Lpin1, which in turn increased the ceramide level in myotubes, directly promoting the expression of serine/threonine protein phosphatase 2A (PP2A) to dephosphorylate AKT that inhibits insulin signaling pathway. Although this study did not involve m6A modification, combined with the fact that *Lpin1* increases half-life in the presence of hepatic hypomethylation (80) and the presence of METTL3 upregulation in PA-induced C2C12 cells (99), we suggested that the reduction of Lpin1 in this study is associated with m6A modification. At present, the role of methylases in skeletal muscle function is still unclear, especially the effect of methylases overexpression or silencing on skeletal muscle function deserve to be explored. Considering that skeletal muscle is the most essential glucose-consuming organ throughout the body, we believe that further exploration is necessary.

The normal expression of FTO is critical for skeletal muscle proliferation and differentiation. FTO ablation affects the mRNA expression of *Pcg-1α* and suppresses the mechanistic target of the rapamycin kinase (mTOR)-PCG-1α pathway, thereby decreasing the mitochondrial energy production and upregulated myogenin expression (100). In such a mechanism, FTO overexpression may enhance mitochondrial energy production leading to skeletal muscle IR, which needs to be explored experimentally. Bravard et al. (101) reported that patients with T2D showed a higher *Fto* level than nondiabetic subjects in skeletal muscle, which was positively correlated with glycosylated HbA1c and blood glucose. The overexpression of FTO in the skeletal muscle myotubes enhances lipogenesis and oxidative stress, leading to skeletal muscle IR. Similarly, the level of m6A modification is negatively associated with skeletal muscle lipid accumulation (102).

Furthermore, a few studies demonstrated that excessive energy causes FTO overexpression, reducing m6A modification. Notably, whether myocytes might secrete inflammatory molecules (103), irrespective of m6A modification, and exacerbate skeletal muscle IR needs further investigation.

## 4 The m6A modification and T2D signaling pathways

The development of T2D is a complex process that cannot be separated from the role of intertwined pathway mechanisms. Herein, we focused on the role of m6A modification in different pathways to explore its profound role in T2D.

### 4.1 PI3K/AKT pathway

Phosphoinositide 3-kinase (PI3K)/AKT pathway regulates energy metabolism homeostasis, cell survival, proliferation, and progression, ensuring organisms’ normal growth. The upstream molecules of the PI3K/AKT pathway are mainly receptor tyrosine kinases, such as the epidermal growth factor receptor (EGFR) family, insulin, and IGF-1 receptor (104); hence, we focused on the pathway of insulin activation in the PI3K/AKT pathway. Initially, insulin binds to the α-subunit of the INSR on the cytosolic membrane, causing phosphorylation of the INSR intracellular β-subunit tyrosine residues. Subsequently, INSR attracts insulin receptor substrates (IRS) and phosphorylates the latter, after which it grips the downstream PI3K p85α subunit SH2 structural domain to bind to the IRS phosphorylated residues, recruiting and activating PI3K p110α subunit. Finally, activated PI3K phosphorylates phosphatidylinositol 4,5 biphosphate (PIP2) to form phosphatidylinositol 3,4,5 triphosphate (PIP3), which recruits and activates AKT in the presence of pyruvate dehydrogenase kinase 1 (PDK1) and mTOR2 (105).

The m6A modification could affect the liver and skeletal muscle PI3K/AKT pathway to influence insulin sensitivity and gluconeogenesis. The upstream and downstream signals of PI3K/AKT pathway were directly affected through alteration of m6A modification. In skeletal muscle, low-abundance m6A modification is positively correlated with hypo-phosphorylation of IRS1, and the phosphorylation of IRS1 is enhanced in skeletal muscle overexpressing *Mettl3 (* 99). Another hepatocytes experiment *in vitro* showed that *Rbm15* silencing improved insulin sensitivity by increasing AKT phosphorylation, whereas *Rbm15* overexpression had the opposite effect (86) (We need to emphasize here again that T2D patients are at low m6A modification overall, but high m6A abundance in the liver). As compared to PI3K/AKT upstream, downstream effector genes are regulated more obviously with m6A modifications, for example, *Foxo1*, *G6pc*, *Fasn* mRNA upregulation in the liver of T2D patients (77), leading to increased glycemia and hepatic steatosis (106). Furthermore, FTO inhibition-treatment increased m6A abundance of *Foxo1* mRNA in the liver, reducing the expression of FOXO1 and gene *G6pc*, which reduces blood glucose by regulating gluconeogenesis (87). Moreover, aberrant energy supply of T2D also led to impaired mTOR and decreased the expression of downstream PCG-1α, thereby affecting the skeletal muscle function (100). Interestingly, the nuclear hormone receptor FOXO1 combines with PGC-1α and regulates gluconeogenesis (107). Notably, Sharabi et al. (108) increased the acetylation of PGC-1α to inhibit hepatic glucose production and alleviate T2D hyperglycemia. This phenomenon suggested that epigenetic modifications explain the mechanisms of the pathway; also, the dynamic and reversible epigenetic modifications could be utilized to modify the key genes of the pathway for disease treatment.

### 4.2 AMPK pathway

AMPK is a critical cellular energy sensor and a crucial regulatory factor for metabolic homeostasis, which could be activated by sensing the changes in the ratio of AMP/ATP and (or) ADP/ATP in the internal environment. AMPK is a multi-isomeric complex comprising α, β, and γ subunits, each of which has 2 or 3 isoforms, and each combined isomer has a unique cellular localization and biological function (109). Moreover, AMPK activation is also affected by PI3K/AKT and intracellular Ca^2+^.

On the one hand, some features of T2D, such as metabolic syndrome and chronic low-grade inflammation, directly impair the activation of AMPK pathway (110). On the other hand, m6A modification also influences AMPK activation, which lead to lipid accumulation in the liver and skeletal muscle. A hepatocyte study *in vitro* showed that LKB1, the upstream of AMPK, is regulated by the methylesterase WTAP and mediates AMPK phosphorylation in an m6A-dependent manner, and when *Wtap* was knocked down, hepatocytes’ AMPK phosphorylation levels were significantly increased (111). In this situation of diminished AMPK activation, downstream adipogenic genes also increase their mRNA stability in an m6A-dependent manner. The high expression of METTL3 and low expression of TYHDF2 in the liver of T2D increased the mRNA stability of *Fasn*, *Acc1*, and *Srebp-1c*, leading to hepatic steatosis and further reducing organ insulin sensitivity (79, 81, 91). Besides, alterations in AMPK also could in turn affect the m6A modification. In skeletal muscle, the knockdown of *Ampkα2* decreases overall m6A abundance, leading to skeletal muscle lipid accumulation (102). Therefore, we suggest that the homeostasis of m6A modification is similar to that of AMPK and is an important modality for the regulation of energy homeostasis in the organism.

### 4.3 JAK/STAT pathway

JAK/STAT is an indispensable signaling node for many cellular processes, a highly conserved signaling pathway in evolution. JAKs (JAK1, JAK2, JAK3, and TYK2) tend to bind to cytokine receptors and hormone receptors, mediated by cytokines and hormones to phosphorylate its tyrosine residues, and then recruit the downstream STATs (STAT1, STAT2, STAT3, STAT4, STAT5a, STAT5b, and STAT6) to alter its conformation (112). Activated STATs enter the cell nucleus to bind to DNA and regulate gene transcription for specific biological processes, such as cell progression, cell differentiation, and lipid metabolism.

Inflammatory cytokines released by adipose tissue in T2D are well-established mediators to exacerbate T2D symptoms (113). These cytokines activate the JAK/STAT pathway in adipose tissue, while hypomethylation of T2D over-activates the JAK/STAT pathway by altering the stability of *Jak* mRNA, and promotes the expression of downstream lipogenic genes, such as C/RBPα/β, PPARγ, and FABP4 (55). Moreover, m6A modification could also regulate the expression of *Suppressor of cytokine signaling 3*, a JAK/STAT downstream gene, which may be associated with adipose tissue inflammation (114). However, further studies on the interactional node of m6A modification and JAK/STAT are still needed.

### 4.4 PPAR pathway

PPARs are members of nuclear transcription factors binding to retinoic X receptors, which could activate upon binding to ligands to regulate lipid metabolism, insulin sensitivity, and energy homeostasis (115). The PPARs superfamily consists of three isoforms: PPARα, PPARγ, and PPARβ/δ. PPARα is mainly located in high energy-consuming organs, such as the liver and skeletal muscle, and regulates energy homeostasis, PPARγ is primarily localized in the adipose tissue and modulates adipocyte formation and insulin sensitivity, and PPARβ/δ is detected in skeletal muscle and adipose tissue, wherein it controls fatty acid metabolism (116).

The PPAR family will increase adipogenesis according to the localization of its members to the liver and adipose tissue, which in turn contributes to the development of T2D. PPARα resembles an energy sensor that perceives circulating fatty acid metabolism, activated by endogenous fatty acid metabolic derivatives in the body (117). Its activation reduces plasma apolipoproteins, enhances β-oxidation of TGs, and increases lipoprotein lipase (LPL), lowering plasma TG and low-density lipoproteins and increasing high-density lipoproteins (118). A previous study showed that hepatic hypomethylation enhances *Pparα* expression, reduces hepatic steatosis, and increases insulin sensitivity (81). Moreover, m6A modification is more complicated in the regulation of PPARγ. In white adipose tissue, hypomethylation contributes to increased *Pparγ* expression, causing lipid accumulation and adipocytogenesis (55, 66); whereas in brown adipose tissue, it decreases *Pparγ* expression, impairing its maturation, which leads to loss of ability to resist obesity and aggravates systemic IR (59). Increasingly evidence suggests that high level of FTO act on the PPAR pathway to enhance adipogenesis, thereby promoting T2D.

## 5 The m6A modification and clinical application

Metformin has been used for >60 years to treat T2D, with clear anti-hyperglycemia and anti-obesity effects. Thus, it is the first-line drug for T2D recommended in the latest American Diabetes Association and European Association for the Study of Diabetes consensus (119, 120). A recent study showed (121) that metformin affects adipogenesis by altering adipose tissue m6A modification to decrease fat mass and alleviate IR *in vivo*. In the *in vitro* experiment, metformin-treated 3T3-L1 cells showed an increased G0/G1 phase ratio and a decreased ratio of S and G2/M phases. Mechanistically, metformin diminishes FTO expression, increasing m6A modification on *Ccnd1* and *cyclin-dependent kinase 2* (*Cdk2*) mRNAs. These manifestations enhance the degradation by TYHDF2, leading to adipocyte differentiation arrest in MCE.

Nowadays, it is proposed that metformin exerts its hypoglycemic effect by activating AMPK; also, m6A modification is involved in the AMPK signaling pathway (122). Thus, exploring how metformin regulates m6A modification might explain the underlying hypoglycemic mechanism.

Glucagon-like peptide-1 (GLP-1) is a gut-secreted hormone that enhances insulin in blood glucose regulation. The combination of GLP-1 and GLP-1 receptors enhances glucose-dependent insulin secretion and inhibits glucagon release; based on this function, several GLP-1 receptor agonists have been developed for T2D treatment (123). In a random controlled trial over three years (124), treatment with GLP-1 receptor agonist, exenatide, revealed that patients with T2D had β-cell biology function. Zhou et al. (125) demonstrated that exenatide reduces the apoptotic effect of oxidative stress on β-cells via augmented *Mettl3* level in primary pancreatic islet cells and NIT-1 cells under H_2_O_2_ treatment. Regrettably, only anti-apoptotic and pro-apoptotic biomarkers were detected, and the regulatory mechanism was not explored.

Thiazolidinedione is an insulin sensitizer and binds to PPARγ to improve insulin sensitivity in the liver, skeletal muscle, pancreas, and other organs to maintain normal blood glucose levels, in contrast to lowering blood glucose directly (126). The common clinical agents include rosiglitazone and pioglitazone. Previous studies (101) have shown that rosiglitazone reduces skeletal muscle *Fto* expression in T2D patients, improving insulin sensitivity in T2D patients. Another study (127) showed that rosiglitazone diminished *Fto* mRNA in subcutaneous adipose tissue and improved glucose utilization in female patients with T2D. However, TZD is now less commonly used clinically, as many safety concerns exist, such as cardiovascular, bone fracture, and bladder cancer risks (128).

To date, only a few studies have addressed pre- and post-treatment methylation changes in T2D. Thus, the drug could modulate the m6A modification in different body organs to affect T2D metabolism, which might be a novel target for T2D treatment.

## 6 Conclusion and prospect

The incidence of the metabolic disease T2D is increasing throughout the world and represents a significant burden for world health. Regrettably, there is still no overall cure for T2D and the molecular pathogenesis of the disease requires full elucidation.

Therefore, we attempted the further understanding of T2D from an epigenetic perspective. We investigated the role of m6A methylation, the most common epigenetic modification in humans, in T2D. Reduced m6A modification was observed in T2D patients, specifically affecting genes involved in glucose and lipid metabolism and pancreatic β-cell biology, thus contributing further insight into the pathophysiology of T2D. Thus, monitoring the m6A modification of critical metabolic genes may represent a new diagnostic target for the assessment of pre-T2D. Furthermore, manipulation of m6A levels may offer a new direction for T2D treatment. Ultimately, novel drugs for the treatment of T2D could be developed to adjust m6A modification, which would further deepen our understanding of the metabolic processes involved in the disease. Taken together, the findings indicated that m6A modification plays a significant role in the pathogenesis of T2D. Further investigation into the key factors and signaling pathways involved in m6A modification, as well as the investigation of potential drugs targeting these modifications, is required to provide new perspectives for T2D treatment.

## Author contributions

Conceptualization, XH and HZ; validation, XH and HZ; investigation, YG, QG, JH and QX; writing—original draft preparation, HZ; writing—review and editing, XH. All authors contributed to the article and approved the submitted version.

## Glossary

**Table:**

T2D	type 2 diabetes
m6A	N6-methyladenosine
IR	insulin resistance
SAM	S-adenosyl methionine
ER	endoplasmic reticulum
TG	triglycerides
IGF1	insulin-like growth factor 1
PDK1	pancreatic and duodenal homeobox 1
ERN1	ER to nucleus signaling 1
XBP-1	X-box protein binding 1
MAFA	MAF bZIP transcription factor A
GSIS	glucose-stimulated insulin secretion
PAX4	paired box 4
GCK	glucokinase
SLC2A2	solute carrier family 2 member 2
SNPs	single nucleotide polymorphisms
FFAs	free fatty acids
JAK	janus kinase
STAT	signal transducer and activator of transcription
C/EBP β	CCAAT enhancer binding protein beta
C/EBPα	CCAAT enhancer binding protein alpha
PPARγ	peroxisome proliferator-activated receptor gamma
FABP4	fatty acid binding protein 4
MCE	mitotic clonal expansion
CCND1	cyclin D1
HFD	high-fat diet
UCP1	uncoupling protein 1
PCG-1α	PPARG coactivator 1 alpha
PRDM16	PR/SET domain 16
RUNX1T1	RUNX1 partner transcriptional co-repressor 1
AMPK	AMP-activated protein kinase
ATG5	autophagy-related 5
ATG7	autophagy-related 7
HIF1-α	hypoxia-inducible factor 1 subunit alpha
GWAS	genome-wide association studies
FASN	fatty acid synthase
SREBP-1c	sterol regulatory element-binding proteins-1c
ACC	acetyl-CoA carboxylase
ACLY	ATP citrate lyase
SCD1	stearoyl-CoA desaturase 1
NLRP3	NOD-like receptor protein 3
DDIT4	damage inducible transcript 4
FOXO1	forkhead box O1
G6PC	glucose-6-phosphatase catalytic subunit
DGAT2	diacylglycerol O-acyltransferase 2
ACVR2a	activin type 2A receptor
PP2A	serine/threonine protein phosphatase 2A
mTOR	mechanistic target of rapamycin kinase
PI3K	phosphoinositide 3-kinase
PDK1	pyruvate dehydrogenase kinase 1
EGFR	epidermal growth factor receptor
INSR	insulin receptor
CDK2	cyclin-dependent kinase 2
GLP-1	glucagon-like peptide-1.
